# Fish Oil Consumption: Its Effects on Bone and Blood Parameters of the Ovariectomized Rat Model of Osteopenia

**DOI:** 10.3390/nu16234046

**Published:** 2024-11-26

**Authors:** Aggeliki Triantafyllou, Pavlos P. Lelovas, Antonis Galanos, Kyriaki Venetsanou, Christina Passali, Anastasia Patsaki, Dimitrios Pitidis, Stavros K. Kourkoulis, Ismene A. Dontas

**Affiliations:** 1 Laboratory for Research of the Musculoskeletal System “Th. Garofalides”, School of Medicine, National & Kapodistrian University of Athens, KAT Hospital, 14561 Athens, Greece; atriantafyllou@med.uoa.gr (A.T.); paulveterin@yahoo.com (P.P.L.); galanostat@yahoo.gr (A.G.); cpassali@gmail.com (C.P.); apatsaki@gmail.com (A.P.); 2 1st Intensive Care Unit, KAT Hospital, 14561 Athens, Greece; kvenetsanou@hotmail.com; 3 Department of Neurology, Evangelismos Athens General Hospital, 10676 Athens, Greece; dpitid@gmail.com; 4 Laboratory of Biomechanics and Biomedical Physics, Department of Mechanics, National Technical University of Athens, 15772 Zografou, Greece; stakkour@central.ntua.gr

**Keywords:** bone mineral density, fish oil, ω-3 fatty acids, ovariectomy, rat

## Abstract

**Background/Objectives**: The beneficial effects of ω-3 fatty acids on the cardiovascular system have been observed in many epidemiological studies; however, their effects on the skeleton and in particular on postmenopausal bone loss appear to vary. The present study’s purpose was to investigate the effects of oral fish oil (rich in ω-3 fatty acids) consumption on bone, plasma, and inflammation parameters in the ovariectomized (Ovx) rat model of osteopenia. **Methods**: Four Groups of ten rats each were separated into Non-Ovx receiving fish oil (2.8 g/kg body weight) (Non-Ovx + FO), Non-Ovx receiving isocaloric corn oil (Non-Ovx + CO), Ovx receiving fish oil (Ovx + FO), and Ovx receiving corn oil (Ovx + CO) daily for 4 months. **Results**: Tibial bone mineral density percentage changes from baseline were +4.09% in Non-Ovx + FO rats versus −2.99% in Non-Ovx + CO rats (*p* NS), and −5.73% in Ovx + FO rats versus −14.12% in Ovx + CO rats (*p* = 0.070), indicating a tendency to protect from bone loss. Fish oil exerted a beneficial effect on bone strength, as shown by significantly increased femoral fracture stress in Ovx + FO, compared to Ovx + CO rats (*p* = 0.05). The plasma’s total cholesterol was significantly reduced in both FO Groups versus the CO Groups (*p* < 0.001), while HDL-cholesterol decreased slightly in both FO Groups, significantly (*p* < 0.001) between Non-Ovx + FO versus Non-Ovx + CO. Interleukin 6 was reduced in both FO Groups, indicating the anti-inflammatory effect of fish oil consumption, which was highly significant (*p* < 0.001) between Non-Ovx + FO versus Non-Ovx + CO. Interleukin 10, TNF-α, and RANKL displayed non-significant changes. **Conclusions**: Among the skeletal and blood parameters studied, several, but not all, demonstrated a mild to significant beneficial effect of four-month fish oil consumption.

## 1. Introduction

Osteoporosis is a systemic skeletal disease characterized by low bone density and microarchitectural disruption of bone tissue, resulting in reduced bone mechanical strength, and increased fracture risk [1]. It is considered one of the main causes of disability and death in advanced age, especially in Western societies where life expectancy has increased [2,3]. The economic burden for the treatment and recovery of patients with osteoporosis and fractures worldwide is worrying, especially with the population increase of those over the age of 65 years. Recent analyses and strategies with the aim to reduce the osteoporosis- and fracture-related clinical and economic burden by 2040 are being conducted in several countries [4,5,6].

Despite significant advances in the pharmaceutical therapy of osteoporosis, limited adherence to therapy has been observed, mainly due to treatment side effects, fear of possible side effects, and secondarily to the cost of therapy [7]. For these reasons, simple, economically feasible changes in eating habits, as well as the addition of nutrients or natural compounds, are the subject of research for possible alternative or supplementary treatment approaches.

In postmenopausal women, estrogen deficiency induces an inflammatory reaction, which may be expressed by increased levels of inflammatory cytokines (IL-1b, IL-6, and TNF-α). These cytokines promote the differentiation and maturation of pre-osteoclasts, and prolong the osteoclast lifespan, resulting in bone loss [8,9].

Omega-3 (ω-3) polyunsaturated fatty acids, which are abundant in fish oil and include eicosapentaenoic acid and docosahexaenoic acid, are known for their anti-inflammatory properties and follow metabolic pathways which appear to reduce the activity of osteoclasts, and thus bone loss [10,11]. The beneficial effects of ω-3 fatty acids in the cardiovascular system have been published in several clinical studies [12,13], while others reported conflicting results [14,15].

Clinical evidence of the beneficial effect of ω-3 fatty acids on skeletal health appears to be inconclusive [16,17,18]. Experimental studies of the effect of ω-3 fatty acids on the skeleton of laboratory animals also have conflicting results [19,20,21,22], which may be due to the duration and dosage of fish oil consumption, and to its individual components.

In the present study, the experimental model chosen was the mature ovariectomized female rat with osteopenia, in order to better simulate the clinical case of women with postmenopausal osteoporosis, who seek nutritional strategies for improving their bone density. Of all of the animal species used for the study of postmenopausal osteoporosis, the rat model above represents significant advantages over, e.g., mice (less-characterized regarding bone loss after ovariectomy, size, and sampling limitations), dogs (differences of estrus cycles compared to humans), and primates (husbandry, ethical, and time limitations) [23].

Most experimental studies concerning non-pharmaceutical interventions are conducted immediately after ovariectomy, which is the phase of rapid bone loss. As this does not represent the majority of clinical cases, where diagnosis of osteopenia occurs long after estrogen depletion when women seek therapy, we selected the rat model of established osteopenia (five months after ovariectomy) for this study. To our knowledge, to date, this is the first study where fish oil administration on the steady state of osteopenia is investigated. Additionally, its consumption for four months, which is one-sixth of a rat’s median lifespan, corresponds to more than a decade of a human life. Since the ovariectomized osteopenic rat does not develop fragility bone fractures, as it occurs in the human condition, the purpose of the present study was to investigate the effects of fish oil oral administration for four months on the bone and blood parameters in ovariectomized, osteopenic mature rats.

## 2. Materials and Methods

### 2.1. Laboratory Animals

The experimental protocol was approved by the Veterinary Directorate of the Prefecture of Athens (experimentation License K/3831/10.06.2008), according to the Greek Laws at the time of the study’s duration (Presidential Decree 160/1991, in compliance with Directive 86/609/EEC and Law 2015/1992, according to the “European Treaty for the protection of vertebrate animals used for experimental and other scientific purposes, 123/1986”). The study took place at the Laboratory for Research of the Musculoskeletal System (LRMS), School of Medicine, National & Kapodistrian University of Athens, KAT Hospital, Greece.

Forty adult (10 months of age) female Wistar rats bred by the LRMS-registered breeding establishment (EL 25 BIO 017) were used. The animals were housed four to a cage, in conventional cages [480 × 375 × 210 mm (L × W × H), Tecniplast, Buguggiate, Italy], under standard laboratory conditions (temperature 19–22 °C, relative humidity 55–65%, 15 complete air changes/hour, 12 h/12 h light/dark cycle).

After conducting individual numbering and baseline body weight measurements (mean body weight 240 ± 13.7 g), the rats were allocated to four Groups, each n = 10, according to their body weight, in order to avoid significant differences between the mean total body weight of each Group. Litter mates were also dispersed among the four Groups, in order to avoid potential genetic differences among Groups. Body weights were monitored every two weeks until the end of the study. Food and water consumption of each cage was measured twice a week.

### 2.2. Ovariectomy

Two of the four Groups underwent an aseptic bilateral ovariectomy (Ovx) at 10 months of age under general anesthesia by ketamine hydrochloride (50 mg/kg body weight i.m., Ketaset, Pfizer Hellas, Athens, Greece) and medetomidine (0.50 mg/kg body weight i.m., Domitor, Elanco Hellas, Athens, Greece), which was reversed by atipamezole (1 mg/kg i.m. Antisedan, Zoetis, Athens, Greece). Pre-operative analgesia was achieved by carprofen administration (4 mg/kg body weight s.c., Rimadyl, Zoetis, Athens, Greece) and chemoprophylaxis by enrofloxacin (10 mg/kg body weight s.c., Baytril, Bayer Animal Health GmbH, Leverkusen, Germany) [24]. The other two Groups underwent the same general anesthetic protocol and were sham-operated. These Groups were necessary in order to have age-matched animals with and without ovariectomy, as well as valid comparisons of potential blood and bone parameter changes between those receiving the fish oil under investigation and those receiving isocaloric corn oil.

### 2.3. Fish Oil and Corn Oil Groups

Administration of fish oil started five months post-ovariectomy, in order to evaluate the potential effect of fish oil on the steady state of established osteopenia, avoiding the early post-ovariectomy period, which is characterized by increased bone remodeling. Two of the four animal Groups received fish oil (FO), and the other two received an isocaloric quantity of corn oil (CO), in order for both the caloric intake, as well as the administration procedure, to be identical in all Groups. The four Groups were as follows:

- Normal rats administered fish oil (Non-Ovx + FO)
- Normal rats administered corn oil (Non-Ovx + CO)
- Ovariectomized rats administered fish oil (Ovx + FO)
- Ovariectomized rats administered corn oil (Ovx + CO)

The comparisons to be conducted were between the following Groups:

- Non-Ovx + FO versus Non-Ovx + CO (evaluation of the effect of each oil on non-ovariectomized rats),
- Ovx + FO versus Ovx + CO (evaluation of the effect of each oil on ovariectomized rats),
- Non-Ovx + FO versus Ovx + FO (evaluation of the ovariectomy effect on rats receiving fish oil), and
- Non-Ovx + CO versus Ovx + CO (evaluation of the ovariectomy effect on rats receiving corn oil).

The capsules OMEGA-3 “700” of the company SOLGAR^®^ (Leonia, NJ, USA) were used, where each capsule contained 700 mg ω-3 fatty acids in 1200 mg fish oil. Fish oil was administered per os at a dose of 2.8 g/kg body weight, which corresponds to a dose of 1.6 g/kg body weight of ω-3 fatty acids. As different authors suggest different doses of ω-3 fatty acids, ranging from 1 g/kg to 1.68 g/kg per day [19,25], we aimed for the higher dose of ω-3 in our study, because of the delayed initiation of administration after ovariectomy. Each capsule was punctured by an insulin needle with the syringe, and the content was withdrawn immediately before administration, so as to avoid potential oxidation. The calculation of the fish oil quantity administered in cm^3^, as well as the corresponding mg and calories, was according to each rat’s body weight. A similar calculation was used for the isocaloric administration of corn oil to the other two rat Groups.

Initially, the per os administration method was used to deliver the oils by mildly restraining the animals with a cloth towel, in order to avoid the daily stress of gavage, and depositing the liquid deep into their mouth with an insulin syringe. Special attention was given to ensure there was no loss of the volume being administered. After the first two weeks, the rats became accustomed to the procedure and did not need further restraining. The administrations took place daily at 9–11 a.m., 6 times a week (excluding Sundays), and for four months.

### 2.4. Blood Sampling—Biochemical Parameters—Cytokines

The primary goal of our study was to investigate the effect of fish oil administration during the phase of steady state bone mass loss—after the rapid bone loss immediately post-ovariectomy—in the mature ovariectomized rat model of osteoporosis. Daily administration of fatty acids for a long period of time may have a negative impact on liver function. Thus, in order to evaluate the safety of fish oil administration, we measured the liver function parameters (transaminases), in addition to investigation of the lipideamic profile (cholesterol, triglycerides), of bone homeostasis (calcium and phosphorus), and of inflammatory actions (cytokines). This was considered necessary in view of the dose and the four-month duration of administration.

Blood samplings were conducted before the administration of fish oil and corn oil, and at the end of the study after four months of administration. The first sampling was conducted under brief inhalation anesthesia (initially induced in a chamber by isoflurane with 4%, maintained by an isoflurane face mask during blood sampling) by retroorbital puncture with a sterile heparinized Pasteur pipette.

The last blood sampling was conducted under general anesthesia (ketamine hydrochloride 50 mg/kg, medetomidine 0.50 mg/kg, both i.m.), immediately prior to euthanasia, via a midline laparotomy and from the posterior vena cava, according to Zervas et al. [24]. The samples were centrifuged at 2000 rpm for 10 min, and plasma was stored at −80 °C until determination of the following parameters, with the corresponding method in parentheses: total cholesterol (CHOD-POD), HDL-cholesterol (immunosuppression), and triglycerides (GPO-POD) were measured using standard laboratory techniques (Liquicolor, Human, Germany). SGOT and SGPT (modified IFCC without pyridoxal phosphate), γ-GT (IFCC), calcium (Arsenazo method), and phosphorus (Molybdate-UV method) were obtained from the company Medicon Hellas S.A. (Athens, Greece). Rat-specific enzyme-linked immunoassay kits were used for the measurement of IL-6, IL-10, tumor necrosis factor alpha (TNFα), and the receptor activator of NFκB Ligand (RANKL). Specifically, the Rat Cytokine Multiplex Immunoassay kit—THREE PLEX, Millipore, Burlington, MA, USA—was used for the measurement of the interleukins and TNF-α; the Rat RANK Ligand Single Plex Immunoassay kit—Lincoplex, Millipore, Burlington, MA, USA—was used for the measurement of RANKL.

### 2.5. Measurement of Bone Mineral Density with Dual-Energy X-Ray Absorptiometry (DEXA)

Measurements of bone mineral density (BMD) were conducted on all rats three times, as follows: (1) before ovariectomy in order to ensure they had normal values, (2) five months later before commencing the administration of FO and CO as baseline measurements, and (3) four months after the administration, in order to assess the effect of the fatty acids. The measurements from these last two time-points were analyzed for our results. The rats were immobilized in order to be scanned by general anesthesia (ketamine hydrochloride 50 mg/kg, medetomidine 0.50 mg/kg, both i.m.). The scans were conducted on a dual-energy X-ray absorptiometry (DEXA) GE Lunar Prodigy Densitometer, using a small animal software. The proximal tibial metaphysis was selected for the evaluation of the BMD of the trabecular bone, as it is a site rich in trabeculae, and it presents rapid bone remodeling, determining a region of interest (ROI) with dimensions of 2 mm × 2 mm, at 3 mm peripheral to the tibial plateau. The in vitro precision of the method is 0.5%. Calibration was performed before scanning each animal Group.

### 2.6. Measurement of Peripheral Quantitative Computerized Tomography (pQCT)

Measurements of the geometrical parameters of all rats’ tibiae were performed by peripheral quantitative computerized tomography (pQCT), Norland-Stratec XCT 2000, immediately after the DEXA measurements, under the same anesthesia. Initially, a scout view of the total tibia was conducted, placing the reference line on the tibial plateau of the knee joint following the five slices that were scanned (Supplementary Materials), and the following two slices were selected to be analyzed: at 4 mm from the plateau (area rich in trabecular bone) and at 15 mm from the plateau (area rich in cortical bone).

### 2.7. Euthanasia, Necropsy, and Tissue Collection

As previously described, following ketamine and medetomidine anesthesia, midline laparotomy incision, and blood samples from the posterior vena cava, the rats were subsequently euthanized with an i.v. injection of sodium thiopental into the vena cava. Necropsy was performed, during which they were carefully examined for possible abnormal findings, such as neoplasia. Successful ovariectomy was confirmed by the absence of ovarian tissue and the observation of hypoplastic uterine horns. The uteri were carefully removed and immediately weighed on a Sartorius handy digital microbalance. The liver and kidneys were also examined and weighed. The right femurs were carefully dissected, cleaned of the surrounding tissues, measured longitudinally with a caliper, wrapped in sterile gauze soaked with normal saline, and stored in −20 °C until biomechanical testing was conducted one week later. This method is the most appropriate for long-term storage of bone samples before their biomechanical testing, without significant deviation from immediate testing [26].

### 2.8. Biomechanical Testing of the Femurs with Three-Point-Bending

Biomechanical testing of the bone strength was conducted ex vivo after the study ended, according to Zervas et al. [24]. The three-point-bending method was selected for its reliability, the brief time during which the bone sample is exposed, and its ease of use. The femur bone samples were left to reach room temperature before being tested. The tests were performed on an MTS 858 Mini Bionix frame (MTS Systems Corp., Eden Prairie, MN, USA) at the Laboratory of Biomechanics and Biomedical Physics of the NTUA. Each bone was placed horizontally on rounded edges at each end of the diaphysis, at a distance of 16 mm. A vertical load was applied in the middle of the diaphysis by using a punch of rounded notch at a displacement rate of 1 mm/min, up to the point of fracture of the bone. At the end of the procedure, the maximal load applied at the fracture was used by the software (TestWorks programmes 4), which created a graph exhibiting the relationship between the load and displacement variables.

### 2.9. Statistical Analysis

Data were expressed as mean ± standard deviation (S.D.) or median, as well as IQR (in case of violation of normality), for continuous variables. The Kolmogorov–Smirnov test was utilized for normality analysis of the parameters. In order to evaluate the changes of the parameters of bone mineral density and blood biochemistry during the four months of the study, the median values of the percentage change from baseline to the end of the study were calculated because of a violation of normality. The comparisons of the percentage changes were evaluated by the Mann–Whitney U-test. The comparisons of the biomechanical testing parameters between Groups were analyzed using the independent samples *t*-test.

All tests were two-sided, and statistical significance was set at *p* < 0.05. All analyses were carried out using the statistical package SPSS v. 21.00 (IBM Corporation, Somers, NY, USA).

## 3. Results

### 3.1. Bone Mineral Density (BMD) Measurements

**Figure 1:**
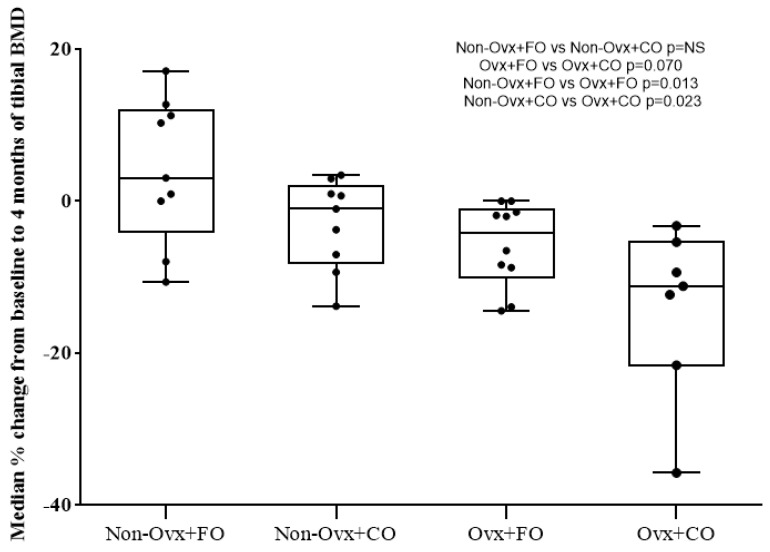
Median percentage % change from baseline (before the administrations) to the end of the study (4 months of administrations) of the proximal tibia BMD measurements. Comparisons and corresponding *p* values: Non-Ovx + FO versus Non-Ovx + CO; N.S. Ovx + FO versus Ovx + CO; N.S. Non-Ovx + FO versus Ovx + FO; *p* = 0.013. Non-Ovx + CO versus Ovx + CO; *p* = 0.023. Abbreviations: BMD: bone mineral density; Ovx: ovariectomized; FO: fish oil; CO: corn oil.

A non-parametric comparison of the percentage change in the median values of the BMD measurements of the proximal tibial metaphysis was conducted from prior until after four months of fish oil (FO) and corn oil (CO) administration to the four experimental Groups. Specifically, the comparisons between pairs of Groups are noted as follows:

- The effect of fish oil or corn oil on Non-Ovx rats: on the rats receiving fish oil (Non-Ovx + FO), the BMD increased by 4.09%, while on those receiving corn oil (Non-Ovx + CO) it reduced by −2.99%. This percentage change from baseline was not statistically significant (*p* = 0.136) (Figure 1).
- The effect of fish oil or corn oil on Ovx rats: on the rats receiving fish oil (Ovx + FO), the BMD reduced by −5.73%, while on those receiving corn oil (Ovx + CO), it reduced by −14.12% (*p* = 0.070), indicating a mild non-statistically significant protective effect of fish oil on bone loss (Figure 1).
- The effect of fish oil on Non-Ovx and Ovx rats: fish oil administration on the rats that were not submitted to ovariectomy (Non-Ovx + FO) resulted in a 4.09% increase in the BMD of their proximal tibial metaphysis, while in the ovariectomized rats (Ovx + FO), there was a −5.73% decrease (*p* = 0.013) (Figure 1).
- The effect of corn oil on Non-Ovx and Ovx rats: corn oil administration on the rats that were not submitted to ovariectomy (Non-Ovx + CO) resulted in a −2.99% decrease of the BMD of their proximal tibial metaphysis, while in the ovariectomized rats (Ovx + CO), there was a −14.12% decrease (*p* = 0.023). Consequently, there was an evident lack of a protective effect of corn oil administration on bone loss (Figure 1).

### 3.2. Bone Peripheral Quantitative Computerized Tomography (pQCT) Measurements

The parameters evaluated by pQCT at 4 mm from the tibial plateau [trabecular content (Trab Cnt), trabecular density (Trab Den), cortical content (Crt Cnt), cortical area (Crt Area)] are available in Supplementary Materials. Pairwise comparisons between Groups revealed statistically significant differences only for the parameters of cortical content and cortical area. These parameters were statistically significantly reduced in the ovariectomized rats receiving fish oil (Ovx + FO) compared to the non-ovariectomized animals receiving fish oil (Non-Ovx + FO), as follows: *p* = 0.03 and *p* = 0.04, respectively.

The respective parameters evaluated by pQCT at 15 mm from the tibial plateau are available in Supplementary Materials. Pairwise comparisons between Groups revealed statistically significant differences only for the parameter cortical content, which was statistically significantly increased in the non-ovariectomized Group receiving fish oil (Non-Ovx + FO) when compared to the ovariectomized Group receiving fish oil (Ovx + FO) (*p* = 0.01). When comparing the Groups of Non-Ovx + FO and Non-Ovx + CO, all of the parameters evaluated were higher, although not statistically significantly, in the animals receiving FO, with a mild trend towards a statistically significant difference for the parameter cortical content (*p* = 0.07).

### 3.3. Bone Biomechanical Testing Measurements

**Figure 2:**
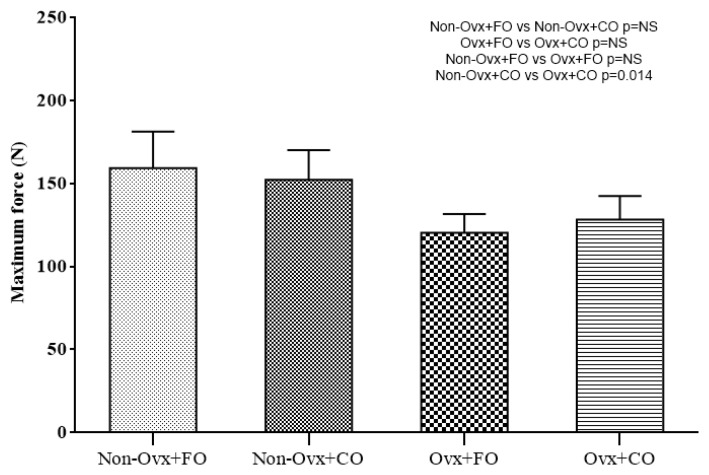
Maximum force.

**Figure 3:**
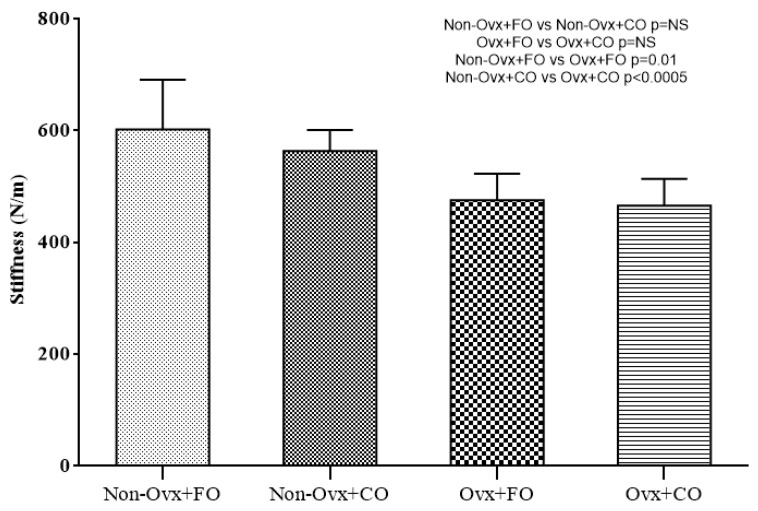
Stiffness.

**Figure 4:**
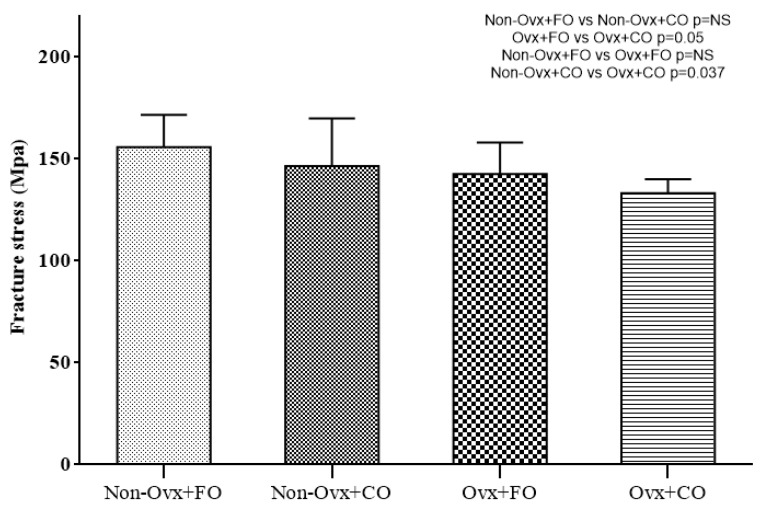
Fracture stress.

Comparisons between the four Groups regarding their biomechanical testing are presented in Figure 2, Figure 3 and Figure 4. The parameters evaluated were maximum force (in N), stiffness (in N/m), and fracture stress (in MPa).

A comparison between the Non-Ovx Groups administered fish oil and corn oil (Non-Ovx + FO versus Non-Ovx + CO) revealed no significant difference in any of the parameters evaluated (Figure 2, Figure 3 and Figure 4).

A comparison between the Ovx Groups administered fish oil and corn oil (Ovx + FO versus Ovx + CO) revealed a statistically significant higher value for the parameter fracture stress (*p* = 0.05) in the Ovx + FO Group (Figure 4).

A comparison between the Groups administered fish oil (Non-Ovx + FO versus Ovx + FO) revealed that the Non-Ovx + FO Group had statistically significant higher values for the parameter stiffness (*p* = 0.01) (Figure 3).

A comparison between the Groups administered corn oil (Non-Ovx + CO versus Ovx + CO) revealed that the Non-Ovx + CO Group had statistically significant higher values for the parameters of maximum force (*p* = 0.014), stiffness (*p* < 0.0005), and fracture stress (*p* = 0.037) (Figure 2, Figure 3 and Figure 4).

### 3.4. Blood Biochemical Parameters and Cytokines

Non-parametric comparisons of the percentage changes of the median values of the blood biochemical parameters and cytokines were made between the sampling values before FO or CO administration and the sampling values four months later, in all Groups.

#### 3.4.1. Total Cholesterol, HDL-Cholesterol, and Triglycerides

**Figure 5:**
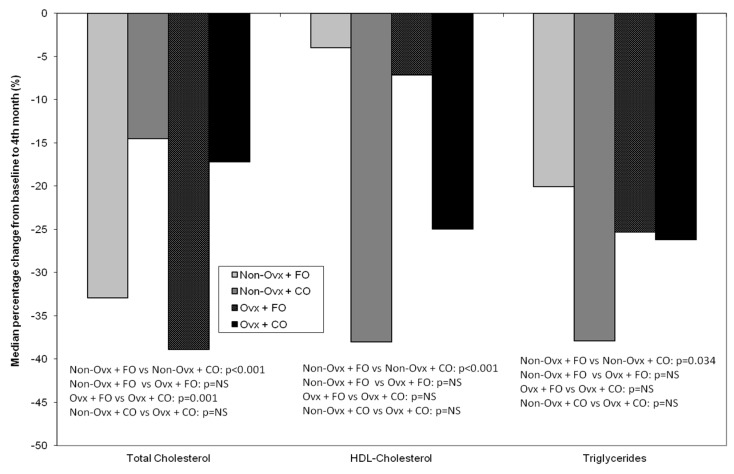
Median percentage % change from baseline (before the administrations) to the end of the study (4 months of administrations) of plasma total cholesterol, HDL-cholesterol, and triglycerides in the four animal Groups: Non-Ovx + FO, Non-Ovx + CO, Ovx + FO, Ovx + CO. Abbreviations: Ovx: ovariectomized; FO: fish oil; CO: corn oil.

- The Groups receiving fish oil (Non-Ovx + FO, Ovx + FO) demonstrated a pronounced decrease in their total cholesterol values, indicating a beneficial effect of FO administration on this parameter (Figure 5). The comparison between Groups Non-Ovx + FO and Non-Ovx + CO was significant (*p* < 0.001), as well as between Groups Ovx + FO and Ovx + CO (*p* = 0.001).
- The comparison of HDL-cholesterol values between Groups Non-Ovx + FO and Non-Ovx + CO was statistically significant (*p* < 0.001), whereas between Groups Ovx + FO and Ovx + CO it was non-significant (*p* value = 0.091). The Groups receiving corn oil (Non-Ovx + CO, Ovx + CO) demonstrated a pronounced decrease in their HDL-cholesterol values, indicating a harmful effect of CO administration on this parameter (Figure 5).
- Triglycerides were decreased in all Groups (Figure 5). The comparison of the non-ovariectomized Groups (Non-Ovx + FO and Non-Ovx + CO) was significant (*p* = 0.034), whereas there was no difference between the ovariectomized Groups Ovx + FO and Ovx + CO.

#### 3.4.2. SGOT/Serum Glutamic-Oxaloacetic Transaminase (AST/Aspartate Transaminase), SGPT/Serum Glutamic-Pyruvic Transaminase (ALT/Alanine Transaminase), and γ-GT (Gamma Glutamyl Transferase)

**Figure 6:**
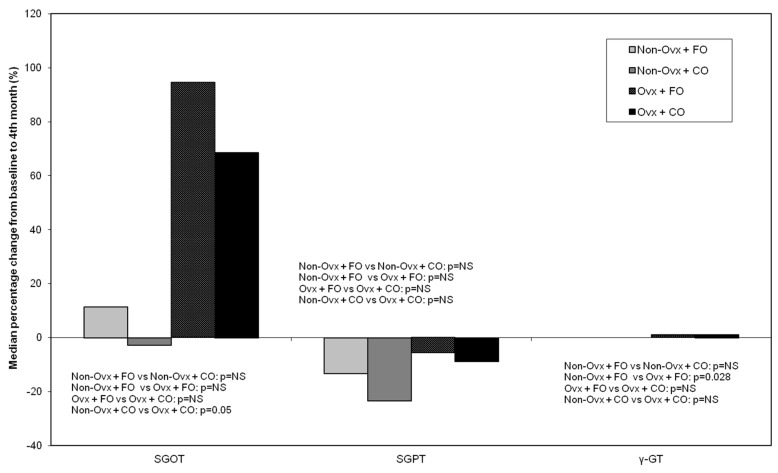
Median percentage % change from baseline (before the administrations) to the end of the study (4 months of administrations) of plasma SGOT, SGPT, and γ-GT in the four animal Groups: Non-Ovx + FO, Non-Ovx + CO, Ovx + FO, Ovx + CO. Abbreviations: SGOT: aspartate transaminase; SGPT: alanine transaminase; γ-GT: gamma glutamyl transferase; Ovx: ovariectomized; FO: fish oil; CO: corn oil.

- SGOT increased in the ovariectomized rat Groups (Ovx + FO and Ovx + CO), with a significant difference (*p* = 0.05) between Groups Non-Ovx + CO and Ovx + CO (Figure 6).
- SGPT decreased in all Groups, with larger percentages in the Non-Ovx Groups, however, without statistical significance (Figure 6).
- γ-GT increased in the ovariectomized rat Groups (Ovx + FO and Ovx + CO), with a significant difference (*p* = 0.028) between Groups Non-Ovx + FO and Ovx + FO (Figure 6).

#### 3.4.3. Calcium and Phosphorus

**Figure 7:**
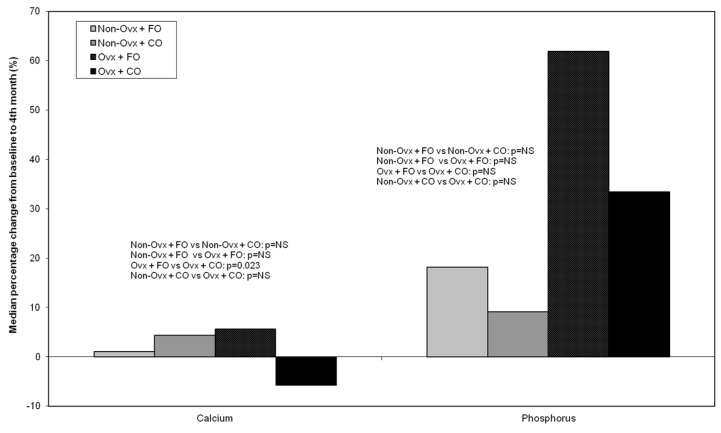
Median percentage % change from baseline (before the administrations) to the end of the study (4 months of administrations) of plasma calcium and phosphorus in the four animal Groups: Non-Ovx + FO, Non-Ovx + CO, Ovx + FO, Ovx + CO. Abbreviations: Ovx: ovariectomized; FO: fish oil; CO: corn oil.

- Calcium values increased during the observation period in all Groups except for Group Ovx + CO. There was a statistically significant difference only between the ovariectomized Groups Ovx + FO and Ovx + CO (*p* = 0.023) (Figure 7).
- Phosphorus values increased during the observation period in all Groups, without statistically significant differences between Groups (Figure 7).

#### 3.4.4. Interleukins 6 and 10 (IL-6, IL-10)

**Figure 8:**
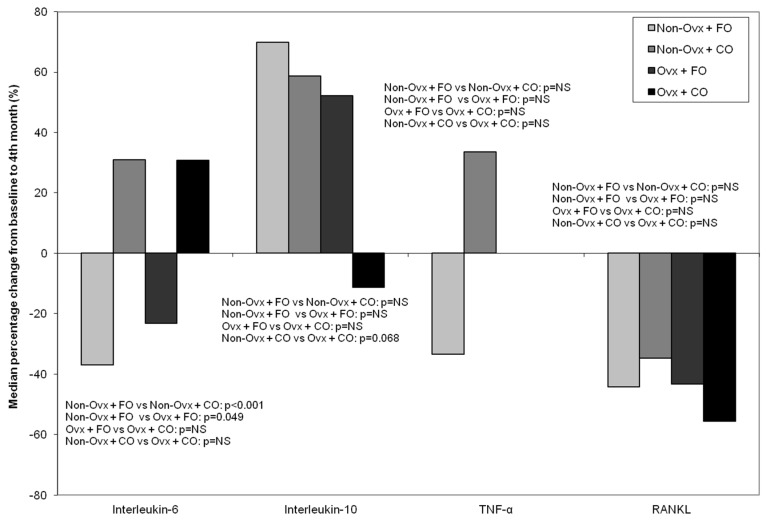
Median percentage % change from baseline (before the administrations) to the end of the study (4 months of administrations) of plasma IL-6, IL-10, TNF-α, and RANKL in the four animal Groups: Non-Ovx + FO, Non-Ovx + CO, Ovx + FO, Ovx + CO. Abbreviations: TNF-α: tumor necrosis factor alpha; RANKL: receptor activator of NFκB Ligand; Ovx: ovariectomized; FO: fish oil; CO: corn oil.

- The Groups that received fish oil (Non-Ovx + FO and Ovx + FO) had a reduction in their IL-6 values, whereas the Groups that received corn oil (Non-Ovx + CO and Ovx + CO) had an increase (Figure 8). The comparison between the non-ovariectomized Groups Non-Ovx + FO and Non-Ovx + CO was statistically significant (*p* < 0.001), as well as between the Groups Non-Ovx + FO and Ovx + FO (*p* = 0.049).
- IL-10 values increased in all Groups except for Group Ovx + CO (Figure 8). The comparison between Groups Non-Ovx + CO and Ovx + CO had a tendency towards significance (*p* = 0.068).

#### 3.4.5. Tumor Necrosis Factor Alpha (TNF-α)

- TNF-α decreased in the non-ovariectomized rats that received fish oil (Non-Ovx + FO), while it increased in those that received corn oil (Non-Ovx + CO), without any statistically significant difference. In the ovariectomized Groups there were no changes at all (Figure 8).

#### 3.4.6. Receptor Activator of NFκB Ligand (RANKL)

- RANKL decreased in all Groups without any statistically significant difference (Figure 8).

## 4. Discussion

The current study evaluated the effect of the administration of fish oil, which is rich in polyunsaturated fatty acids, on the mature female ovariectomized rat model during the steady state of osteopenia, for a total of four months. Isocaloric corn oil was administered for comparison, in order for all animal Groups to be receiving the same calories in addition to their rat diet. This was necessary, since potential weight gain—which increases bone mineral density (BMD)—in the animals receiving fish oil may have confounded the results if only fish oil was administered.

A post hoc power analysis using the median % change from baseline to the end of the study of the BMD proximal tibia measurements between Ovx + FO versus Ovx + CO (−5.73% versus −14.12%), with a = 0.05 and n = 10, revealed a power of 91%. Our evaluation of BMD at the proximal tibial metaphysis revealed that the administration of fish oil had a statistically significant beneficial effect on the Non-Ovx compared to Ovx animals (*p* = 0.013). A similar result was noted by Matsushita et al. [19], who demonstrated that fish oil administration on mature rats, beginning prior to ovariectomy, had a significant beneficial impact on their bone mineral content (BMC) when compared to Ovx rats fed a normal diet. In our study, the beneficial effect of fish oil on the BMD of the proximal tibial metaphysis when compared to corn oil was apparent, but not statistically significant in the Ovx rats (a reduction of −5.73% versus −14.12%, *p* = 0.07). This may be due to the small number of animals used, to the duration of administration (4 months), or to the time-point selected for the commencement of fish oil administration. Furthermore, similarly to our study, a study on fish oil supplementation in ovariectomized mice for six months documented significantly reduced BMD loss in the fish oil-fed mice compared to the corn oil-fed mice, which was attributed to inhibition of the production and activation of osteoclasts by fish oil [27]. Another study also demonstrated a beneficial impact of ω-3 fatty acid administration on the BMC of the femur in growing rats [28]. Our specific evaluation of bone quality by pQCT at two bone sites, rich in trabecular and cortical bone, respectively, indicated changes between the Non-Ovx and Ovx animals receiving the same supplement, which highlights the effect of ovariectomy, rather than that of the oil administered.

Our results on the bone strength parameters evaluated by the three-point-bending method demonstrated that fish oil administration to the Ovx rats produced a statistically significant increase in their fracture stress compared to the Ovx animals receiving corn oil. Furthermore, the absence of a statistically significant difference between Non-Ovx + FO and Ovx + FO for the parameters of maximum force and fracture stress indicated a beneficial effect of FO on bone mechanical strength after ovariectomy, while the presence of a statistically significant difference for the same parameters between the Non-OVX + CO when compared to OVX + CO indicated that the administration of CO was not able to protect bone mechanical strength after ovariectomy.

There is substantial evidence supporting that an adverse lipid profile is related to a higher risk of osteoporotic fractures [29,30]; for this reason, we assessed the lipidaemic profile of the rats by evaluating their total cholesterol, HDL-cholesterol, and triglycerides. The biochemical results of our study are in agreement with studies indicating that fish oil consumption reduces plasma triglycerides [12,31]. More specifically, the comparison of total cholesterol values revealed a statistically significant reduction in the Groups receiving fish oil when compared to those receiving corn oil (*p* < 0.001 both Non-Ovx and Ovx, Figure 5). However the reduction of HDL-cholesterol was less in the rat Groups supplemented with fish oil, with a significant difference between the Non-Ovx + FO and Non-Ovx + CO Groups (−4% versus −38%, *p* < 0.001, Figure 5). This is in agreement with observations made in humans that, contrary to ω-6 polyunsaturated fatty acids, fish oil does not reduce HDL-cholesterol [12]. Additionally, the percentage change from the baseline of triglycerides values was reduced in all Groups, irrespective of fish or corn oil supplementation.

In the present study, calcium percentage changes from baseline increased in all Groups except for the Ovx + CO Group. A comparison between Ovx animals receiving fish or corn oil revealed a statistically significant difference (*p* = 0.023). This is in agreement with another study conducted by Moselhy et al. [32], where cod liver oil supplementation had the same impact on calcium levels as oestrogen administration in the Ovx rat model of osteoporosis. The authors concluded that both oestrogen and cod liver oil administration in Ovx rats increased the calcium deposit in their femurs, probably through stimulation of bone formation and calcification.

We investigated IL-6 as an inflammatory cytokine, which was decreased in the Groups receiving fish oil, while the decrease was more prominent in the Non-Ovx animals when compared to the Ovx. The other inflammatory cytokine investigated, TNF-α, was also reduced in the Non-Ovx + FO animals, albeit non-significantly, while RANKL expression was decreased in all Groups. Our results are in agreement with studies that have documented a reduction of IL-1b, IL-6, and TNF-α in cell cultures of mice after the addition of fish oil, and also following 8 weeks of ω-3 consumption in male rats [27,33]. A non-significant increase of the anti-inflammatory cytokine IL-10 was noted in both FO Groups, which reinforces the concept of an anti-inflammatory effect, which has also been reported by Xia et al. [33]. As we noted, given the reduction of the inflammatory cytokine IL-6 and the increase of the anti-inflammatory cytokine IL-10 in the fish oil-administered Groups (both non-ovariectomized and ovariectomized rats), and given the fact that bone density loss has been considered to be inflammation-related, we assume that fish oil exerted an anti-inflammatory effect. These experimental results should be interpreted with caution if one wishes to extrapolate data from animal studies to humans.

The present study, if it had included additional animal Groups with higher and lower doses of fish oil, may potentially have yielded more distinct and significant results of its effect. This may be considered a limitation, however, the use of more animals without it being absolutely necessary, according to the principle of reduction of Russell and Burch, is to be avoided for animal welfare reasons [34]. A longer administration period may also be considered desirable to potentially produce clearer results. Other similar studies investigating the effects of fish oil on rats vary from 2–16 weeks of administration [19,20,22,28,32,35]; we selected the 16-week (4 month) administration period. It could be considered that an additional limitation was the non-inclusion of animal Groups with and without ovariectomy and without any oil administration. This was decided during the study design when considering the most appropriate “control” Groups for the fish oil-administered rats, both ovariectomized and not. As fish oil contains a considerable caloric value, it was decided that the most appropriate “control” Groups should be supplemented with an isocaloric oil, i.e., corn oil, which was also applied in other studies [21,27]. The option of additional Groups, ovariectomized and non-ovariectomized rats with no administration, was decided against, in order to comply with the above-mentioned principle of reduction [34].

## 5. Conclusions

Fish oil consumption exerted a beneficial effect on the BMD of both Ovx and Non-Ovx Groups, although non-significantly. It also improved the biomechanical parameters of Ovx rats that consumed fish oil. Furthermore, it improved the lipidaemic profile by reducing the total cholesterol and triglyceride levels in both fish oil-administered Ovx and Non-Ovx Groups. It induced a significant reduction of the inflammatory cytokine IL-6, which was more prominent in the Non-Ovx compared to the Ovx Group. It also produced a non-significant increase of the anti-inflammatory cytokine IL-10 in both fish oil-administered Groups. Overall, from our study’s results, it appears that fish oil supplementation that occurs late during the steady state of bone loss had a beneficial effect on several of the parameters studied. Further studies are needed in order to translate these results to the clinical situation of postmenopausal women.

## Data Availability

The raw data from which the results are presented in this study are available on request from the corresponding author. The raw data are not publicly available due to their archiving in the Laboratory for Research of the Musculoskeletal System’s files.

## Supplementary Materials

The following supporting information can be downloaded at: https://www.mdpi.com/article/10.3390/nu16234046/s1, Table S1: Comparison between Groups of the median values at the end of the study (4 months of administrations), the interquartile range and *p* value of pQCT parameters measured at 4 mm from the tibial plateau. Table S2: Comparison between Groups of the median values at the end of the study (4 months of administrations), the interquartile range and *p* value of pQCT parameters measured at 15 mm from the tibial plateau.
