# Supplementary material for: Fish Oil Consumption: Its Effects on Bone and Blood Parameters of the Ovariectomized Rat Model of Osteopenia
Source: Nutrients. 2024 Nov 26;16(23):4046. doi: 10.3390/nu16234046 (PMC11643879; doi:10.3390/nu16234046)
Supplement: Supplementary file 1 [file nutrients-16-04046-s001.zip › nutrients-3325016-supplementary.pdf]

Supplementary Materials

Bone peripheral Quantitative Computerized Tomography (pQCT) measurements

The parameters evaluated by pQCT at 4 mm from the tibial plateau [trabecular content (Trab Cnt), trabecular density (Trab Den), cortical content (Crt Cnt), cortical area (Crt Area)] are shown in Supplementary Table 1. Pairwise comparisons between Groups revealed statistically significant differences only for the parameters Cortical Content and Cortical Area. These parameters were statistically significantly reduced in the ovariectomized rats receiving fishoil (Ovx + FO) compared to non-ovariectomized animals receiving fishoil (Non-Ovx + FO): p=0.03 and p=0.04 respectively.

Supplementary Table S1. Comparison between Groups of the median values at the end of the study (4 months of administrations), the interquartile range and p value of pQCT parameters measured at 4 mm from the tibial plateau.

**Parameter**	**Median**	**IQR**	**Median**	**IQR**	**p value**
**Group**	**Non-Ovx + FO**		**Non-Ovx + CO**		
Trab Cnt (mg/mm)	10.08	(-22.82, 35.42)	0.00	(-20.07, 33.66)	0.89
Trab Den (mg/mm^2)	-4.44	(-9.11, 0.28)	-1.78	(-6.25, 0.00)	0.61
Crt Cnt (mg/mm)	-0.62	(-3.62, 8,68)	-1.98	(-7.39, -0.17)	0.37
Crt Area (mm^2)	1.92	(-4.89, 8.61)	-1,02	(-8.17, 4.09)	0.42
**Group**	**Non-Ovx + FO**		**Ovx + FO**		
Trab Cnt (mg/mm)	10.08	(-22.82, 35.42)	-6.74	(-33.11, 15.00)	0.42
Trab Den (mg/mm^2)	-4.44	(-9.11, 0.28)	-10.69	(-22.73, -0.15)	0.32
Crt Cnt (mg/mm)	-0.62	(-3.62, 8,68)	-14.32	(-19.39, -0.81)	**0.03**
Crt Area (mm^2)	1.92	(-4.89, 8.61)	-14.55	(-24.01, -1.61)	**0.04**
**Group**	**Ovx + FO**		**Ovx + CO**		
Trab Cnt (mg/mm)	-6.74	(-33.11, 15.00)	2.46	(-20.06, 36.25)	0.44
Trab Den (mg/mm^2)	-10.69	(-22.73, -0.15)	0.23	(-17.04, 12.87)	0.28
Crt Cnt (mg/mm)	-14.32	(-19.39, -0.81)	0.97	(-16.39, 16.44)	0.16
Crt Area (mm^2)	-14.55	(-24.01, -1.61)	-0.14	(-17.99, 15.90)	0.23
**Group**	**Non-Ovx + CO**		**Ovx + CO**		

Trab Cnt (mg/mm)	0.00	(-20.07, 33.66)	2.46	(-20.06, 36.25)	0.80
Trab Den (mg/mm^2)	-1.78	(-6.25, 0.00)	0.23	(-17.04, 12.87)	0.51
Crt Cnt (mg/mm)	-1.98	(-7.39, -0.17)	0.97	(-16.39, 16.44)	0.96
Crt Area (mm^2)	-1,02	(-8.17, 4.09)	-0.14	(-17.99, 15.90)	0.57

Abbreviations: IQR: interquartile range; Trab Cnt: trabecular content; Trab Den: trabecular density; Crt Cnt: cortical content; Crt Area: cortical area; Ovx: ovariectomized; FO: fishoil; CO: cornoil.

The respective parameters evaluated by pQCT at 15 mm from the tibial plateau are shown in Supplementary Table 2. Pairwise comparisons between Groups revealed statistically significant differences only for the parameter Cortical Content, which was statistically significantly increased in the non-ovariectomized group receiving fishoil (Non-Ovx + FO) when compared to the ovariectomized Group receiving fishoil (Ovx + FO) ($p=0.01$). When comparing the Groups Non-Ovx + FO and Non-Ovx + CO, all the parameters evaluated were higher, although not statistically significantly, in the animals receiving FO, with a mild trend towards statistically significant difference for the parameter Cortical Content ($p=0.07$).

Supplementary Table S2. Comparison between Groups of the median values at the end of the study (4 months of administrations), the interquartile range and p value of pQCT parameters measured at 15 mm from the tibial plateau.

**Parameter**	**Median**	**IQR**	**Median**	**IQR**	**p value**
**Group**	**Non-Ovx + FO**		**Non-Ovx + CO**		
Tot Area (mm^2)	15.79	(-19.17, 109.52)	-9.52	(-65.05, 55.77)	0.37
Crt Cnt (mm/mg)	4.72	(1.68, 6.34)	0.27	(-3.23, 3.94)	0.07
Crt Area (mm^2)	6.90	(-1.79, 7.69)	0.00	(-2.50, 2.68)	0.32
Peri C (mm)	7.61	(-10.13, 44.47)	-5.01	(-39.19, 24.57)	0.42
**Group**	**Non-Ovx + FO**		**Ovx + FO**		
Tot Area (mm^2)	15.79	(-19.17, 109.52)	25.36	(0.00, 37.95)	0.96
Crt Cnt (mm/mg)	4.72	(1.68, 6.34)	-0.75	(-5.25, 2.27)	**0.01**
Crt Area (mm^2)	6.90	(-1.79, 7.69)	0.00	(0.00, 3.81)	0.54
Peri C (mm)	7.61	(-10.13, 44.47)	11.91	(0.00, 17.46)	0.96

Group	Ovx + FO		Ovx + CO		
Tot Area (mm^2)	25.36	(0.00, 37.95)	26.67	(-7.92, 57.21)	0.80
Crt Cnt (mm/mg)	-0.75	(-5.25, 2.27)	1.33	(-3.79, 2.84)	0.65
Crt Area (mm^2)	0.00	(0.00, 3.81)	2.00	(-2.88, 6.91)	0.80
Peri C (mm)	11.91	(0.00, 17.46)	23.93	(-4.05, 37.53)	0.38
**Group**	**Non-Ovx + CO**		**Ovx + CO**		
Tot Area (mm^2)	-9.52	(-65.05, 55.77)	26.67	(-7.92, 57.21)	0.51
Crt Cnt (mm/mg)	0.27	(-3.23, 3.94)	1.33	(-3.79, 2.84)	0.80
Crt Area (mm^2)	0.00	(-2.50, 2.68)	2.00	(-2.88, 6.91)	0.80
Peri C (mm)	-5.01	(-39.19, 24.57)	23.93	(-4.05, 37.53)	0.51
